# Intention to Purchase Eco-Friendly Handcrafted Fashion Products for Gifting and Personal Use: A Comparison of National and Foreign Consumers

**DOI:** 10.3390/bs13020171

**Published:** 2023-02-14

**Authors:** Dindin Saepudin, Alireza Shabani Shojaei, Belem Barbosa, Isabel Pedrosa

**Affiliations:** 1 Coimbra Business School, Polytechnic Institute of Coimbra, 3045-601 Coimbra, Portugal; 2 School of Economics and Management, University of Porto, 4200-464 Porto, Portugal; 3 Research Unit on Governance, Competitiveness and Public Policies (GOVCOPP), Center for Economics and Finance at UPorto (cef.up), School of Economics and Management, University of Porto, 4200-464 Porto, Portugal

**Keywords:** purchase intention, fashion motivation, perceived value, price perception, recycled cloth, traditional cloth, batik, artisan fashion products

## Abstract

This study aims to examine consumer intention to purchase eco-friendly, handcrafted fashion products made from upcycled clothing and traditional Indonesian batik fabric. Data were collected via an online questionnaire with 289 participants, including both Indonesian and non-Indonesian consumers. The hypotheses were tested using structural equation modeling in SmartPLS 3. The results showed that fashion motivation and perceived value positively impacted the intention to purchase this type of product for personal use and for gifting. The perceived price had a positive effect on purchase intention for gifting. Altruistic motivations affected attitudes but not purchase intentions. Differences were identified between national and foreign consumers regarding the impact of price perception on attitudes and personal purchase intentions. The study provides practical implications for small businesses, artisan crafts, and entrepreneurs.

## 1. Introduction

The fashion industry is widely regarded as one of the most polluting industries in the world, with annual carbon emissions of 2.1 billion tons, constituting 4% of the world’s total greenhouse gas emissions [1]. The production of short-lived, low residual value products [2] results in an estimated 11 million tons of textile waste. Reuse is emphasized as the most effective way to reduce the environmental impact of the fashion industry [3]. Consequently, in recent years, eco-friendly fashion has become a popular solution to these environmental issues [4].

Recycling to reduce waste is a common practice in producing contemporary fashion products by designers and artisans. The role of artisans in mitigating the environmental impact of the fashion industry is becoming increasingly important, as handcrafted goods are growing in popularity, with Etsy’s handmade crafts doubling their revenue in 2020 and 39% growth in sales during the first nine months of 2021 [5]. The sustainable fashion market, valued at over $6.5 billion, is projected to reach $10.1 billion by 2025 and $15 billion by 2030. The Asia Pacific region accounts for 36% of the global ethical fashion market, the largest share, and 73% of millennials indicate they are willing to pay more for sustainable brands [6], leading to an influx of eco-friendly businesses [7]. Despite growing consumer awareness of recycled fashion products’ environmental and ethical benefits [8,9,10], the recycling of used clothing remains low [11]. Fashion artisans play a crucial role in preserving cultural traditions and the symbolic significance of traditional practices and materials [12], exploring cultural traditions, old techniques, and traditional materials such as cloth [13]. Despite the importance of recycling for reducing textile waste and promoting sustainability, research on the adoption of these products is limited.

Business success is contingent upon the ability to attract consumers from international markets. Research has indicated that individuals from different countries exhibit distinctive decision-making styles [14,15] and purchasing behaviors [16,17] in the context of consumer purchase behavior. However, the current literature on consumer behavior toward handcrafted fashion products is lacking, particularly concerning international perspectives and the examination of eco-friendly alternatives. While prior studies have explored the reasons behind purchasing eco-friendly products, there has been limited attention paid to the purchase intentions of handcrafted fashion products for personal use and gifting. This study aims to bridge this research gap by investigating the factors influencing consumers’ intention to purchase eco-friendly handcrafted fashion products.

Furthermore, previous research has analyzed the motivations behind purchasing second-hand products and used clothing from an economic and recreational standpoint, but the role of fashion motivation remains unexamined. Ferraro, et al. [18] argued that there is insufficient research on the impact of ‘fashionability’ as a motivation for purchasing second-hand goods. Additionally, literature reviews have indicated a substantial amount of research conducted on consumer behavior within specific cultures, but a shortage of comparative studies that examine consumer behavior across cultures [19,20]. To the best of the authors’ knowledge, a comparative study has yet to be conducted that examines the intention to purchase eco-friendly handcrafted fashion products among national and foreign consumers.

This study examines the key factors that influence consumer purchase intention towards eco-friendly handcrafted fashion products made of both recycled and traditional fabrics. A comprehensive literature review is presented in the next section, and hypotheses are formulated to explain the purchase intention for personal use and gifting of such products. Furthermore, this article includes an empirical investigation that compares the results between national and international consumers.

## 2. Literature Review and Hypotheses Development

This section synthesizes the key contributions from the literature on the purchase intention of eco-friendly handcrafted fashion products and provides a basis for the development of research hypotheses.

The notion of intention originates from psychology and refers to the likelihood of an individual engaging in a particular action [21]. In the context of consumer behavior, purchase intention is defined as the probability that consumers will follow through with a purchasing behavior, which is distinct from purchase desire [22]. The purchase intentions of individuals are expected future behaviors that reflect the anticipation or plan to make a purchase based on their perceptions, motivations, and attitudes [23,24].

### 2.1. Fashion Motivation

Consumers are becoming increasingly cognizant of sustainability and are eager to identify and purchase products that align with their values, including their desire to protect the environment. Sustainability is expected to enhance the value of fashion products [25] and shape consumers’ attitudes toward fashion consumption [26]. Fashion motivation is linked to personal expression through clothing, whether standing out with unique and authentic pieces, staying on trend, or creating a unique style [27,28]. Sproles and Burns [29] define fashion as “a style of consumer product…that is temporarily adopted by a recognizable segment of individuals” (p.4). In accordance with the definition of fashion stated by [18], in the context of this research, fashion refers to the extent to which customers view eco-friendly handcrafted products (made from upcycled clothing and traditional fabric) as fashionable. In contrast to the past, when the use of second-hand clothing was driven by financial necessity and considered undesirable [30], second-hand clothing and eco-friendly handcrafted products made from upcycled clothing and traditional fabric have now become popular and desirable fashion choices [18,27]. According to Guiot and Roux [31], the appeal of second-hand clothing lies in its authenticity and unique vintage aspect.

Fashion motivation has a significant impact on consumers’ perceived benefits of more sustainable clothing, such as recycled or reused products [10]. The literature also highlights that sensory dimensions such as touch, aesthetics, and emotions, are the most important factors in fashion motivation and contribute to consumers’ attitudes. Hence, it is expected that:

**H1:**  *Fashion motivation positively influences consumer attitudes toward eco-friendly handcrafted products.*

Eco-friendliness, style, quality, and trendiness are considered crucial factors in fashion motivation [32]. Handcrafted products are perceived as especially desirable as gifts. In fact, consumers’ experiences with authenticity, such as handcrafted and bespoke products, lead to higher average sales than traditionally manufactured products, particularly for gifts [33,34]. Frizzo et al. [35] found that consumers’ experiences with authentic handcrafted products play a crucial role in shaping their purchase intention. Accordingly, it is expected that:

**H2:**  *Fashion motivation is positively related to the intention to purchase eco-friendly handcrafted products for gifting.*

Handcrafted products are also becoming increasingly appealing as a fashion motivation. Rahman and Kharb [36] found that fashion innovativeness is particularly valued by young consumers when purchasing clothing. Furthermore, handcrafted products that incorporate ethnic identities and cultural symbols are growing in popularity in fashion, particularly to maintain the authenticity of their country of origin [37]. The literature also highlights that fashion motivation for ethnic identity plays an important role in explaining purchase intentions and is meaningful for individuals who use it. Boncinelli et al. [38] found that the most relevant attribute for consumer purchases of a product, especially for personal use and consumption, is its place of origin. Consumers tend to be more likely to purchase these fashion products for personal use due to their authentic and unique nature [33]. Based on these insights, it is expected that:

**H3:**  *Fashion motivation is positively related to the intention to purchase eco-friendly handcrafted products for self-use.*

### 2.2. Perceived Value

Zeithaml [39] defined perceived value as “the consumer’s overall assessment of the utility of a product based on perceptions of what is received and what is given”. According to Ginsberg and Bloom [40], when given a choice, consumers tend to prioritize product attributes over environmental impact, making it important for companies to employ effective green marketing strategies to enhance the perceived value of their products by highlighting their environmental responsibility [41,42].

The consumer’s satisfaction and desire for a product can be gauged through perceived value, especially for sustainable products. Sustainable fashion trends have the potential to provide profitable value for desired products [43]. Perceived value plays a crucial role in the consumer decision-making process, as consumers are likely to choose products with a higher perceived value [44]. Firms can increase consumers’ likelihood of purchase their products by improving their perceived value [45].

Yu and Lee [46] found that consumers’ perceived value of a product’s green and environmental aspects significantly impacts their behavior, particularly regarding positive attitudes and purchase intentions. Chen and Chang [41] found that perceived value has a positive impact on attitude. Yu and Lee [46] demonstrated that perceived value, including green, emotional, and aesthetic values, has a significant positive impact on consumer attitudes toward upcycled products. Sustainability provides added value benefits by extending the product life span [43] and the life cycle of used clothing consumption [47]. Based on this, it is expected that:

**H4:**  *Perceived value is positively related to consumer attitude toward eco-friendly handcrafted products.*

Sustainable handcrafts are becoming more popular, as consumers value products made by passionate and skilled artisans. This is reflected in their perceived value of these products, particularly when gifting to loved ones [48]. Diddi and Yan [49] argue that consumers are highly engaged in sustainable post-clothing consumption, especially when they possess clothing repair skills, which leads to a higher perceived value and intention to purchase sustainable fashion. Perceived value can impact consumer purchase intentions, with a low perception of value leading to a decrease in consumer purchasing decisions [50].

Based on previous literature, perceived value is a significant predictor of customer purchase intention [51] and a direct contributor to purchase intention in the context of eco-friendly products [42,52,53]. Yadav and Pathak [42] found that perceived value positively impacts consumer green purchase intention and behavior. Several researchers have emphasized that perceived value is the most important criterion in consumer purchase intention when gifting to others [48,54]. In line with these contributions, it is expected that:

**H5:**  *Perceived value positively related to the intention to purchase eco-friendly handcrafted products for gifting.*

The perceived value of a product has a significant impact on consumers’ preferences and experiences regarding sustainable products. According to Rahman and Gong [55], sustainable fashion design, particularly personalized items, can effectively encourage consumers to purchase and use these products [55]. Environmentally friendly products have important information, value, and quality features, and strongly influence perceived value and purchase intention [56]. Bulut et al. [57] found that prior positive experiences with green products often lead to high levels of purchase intention. The literature also highlights the role of credibility in perceived value, particularly among post-millennial consumers. Once they purchase eco-friendly handcrafted products for personal use, they are more likely to recommend them to others. Based on these findings, it is predicted that:

**H6:**  *Perceived value is positively related to the intention to purchase eco-friendly handcrafted products for self-use.*

### 2.3. Price Perception

Price is a significant factor that affects consumer preferences for sustainable products and their willingness to purchase [4]. Consumers’ purchasing decisions are commonly based on their perceived price and overall opinion of the actual price of a product [58,59]. Rahman and Koszewska [43] emphasized that price plays a crucial role in sustainable fashion, particularly in the evaluation of clothing by younger consumers and females. Despite consumers’ willingness to pay a premium price for sustainable products [34,48], Levrini and Santos [60] found that price perceptions affect consumers’ assumptions.

Studies show that most customers perceive upcycled fashion products as overpriced [61,62]. Niinimaki, et al. [63] suggested that consumers should view fashion as a functional product and be willing to pay more for environmentally conscious products. However, limited research exists on the impact of price perception on consumers’ attitudes toward eco-friendly handcrafted products. The literature suggests that sensory impressions, additional information, and product quality can impact consumers’ decisions on price, making price perception a critical factor in consumers’ attitudes toward sustainable fashion products [64]. As a result, it is expected that:

**H7:**  *Price perception is positively related to consumer attitude toward eco-friendly handcrafted products.*

Most consumers prioritize obtaining the lowest price, particularly when product quality is similar [65,66]. This has led to studies indicating a positive relationship between price and purchase intention [59,65,67,68]. Despite high price perceptions of eco-friendly products, consumers still consider purchasing sustainable and secondhand fashion items [26,64]. Price positively impacts consumer intention to purchase fashion products [69], and is a critical characteristic considered when purchasing environmentally friendly products [70]. In gift-giving situations, price is an important indicator of consumers’ purchasing decisions [38,71]. Thus, it is expected that:

**H8:**  *Price perception is positively related to the intention to purchase eco-friendly handcrafted products for gifting.*

Additionally, price perception is a predictor of consumer purchase intention for eco-friendly products. Despite price being a crucial factor in purchasing decisions, consumers believe that higher-priced eco-friendly products for personal use are of higher quality and have a greater positive impact on the environment [72]. Thus, it is expected that:

**H9:**  *Price perception is positively related to the intention to purchase eco-friendly handcrafted products for self-use.*

### 2.4. Altruistic Motivation

Altruism is a value that can influence consumer behavior and the intention to purchase sustainable products, which benefit the environment and society. Research on altruistic motivation in sustainable product purchasing has produced inconsistent results. While some studies show a positive relationship between altruism and green behavior [73,74], others [75] suggest that high levels of altruism do not necessarily result in eco-friendly product purchasing. In the context of sustainable clothing, Prakash, et al. [76] found that young Indian customers with altruistic values had a positive attitude towards eco-friendly products. Jacobs et al. [77] discovered that an altruistic motivational preference for sustainable clothing positively affects consumer attitudes, as consumers prioritize sustainability benefits, such as durability, over “fashionability”. Mishal, et al. [78] also found that consumers with positive attitudes toward eco-friendly products, who actively participate in waste reduction and recycling, have a positive attitude towards sustainable products. Pop et al. [79] confirmed that altruistic motivations have a significant impact on consumer attitudes toward green products. Based on these findings, it is hypothesized that:

**H10:**  *Altruistic motivation is positively related to consumer attitudes toward eco-friendly handcrafted products.*

The study by Barbarossa and Pelsmacker [80] found that the motivation to care for the environment has a significant impact on the intention to purchase eco-friendly products among green consumers. Pro-environmental consumers were found to have a stronger altruistic motivation on purchase intention. Dhaliwal et al. [81] also emphasized that altruistic motivation plays a crucial role in consumer behavior, especially for gifting. Producing handcrafted products can increase consumers’ purchase intention, especially when gifting to loved ones [48]. Hence, it is expected that:

**H11:**  *Altruistic motivation is positively related to the intention to purchase eco-friendly handcrafted products for gifting.*

Additionally, Prakash, Choudhary, Kumar, Garza-Reyes, Khan and Panda [76] stated that consumers with altruistic values are more likely to buy products with eco-friendly packaging. Lundblad and Davies [82] found that consumers are motivated to consume sustainable fashion as it helps reduce waste and support the environment. Consumers self-designate themselves to support the environment by purchasing second-hand or recycled clothes for personal use [82]. It is suggested in the literature that consumers will continue to purchase sustainable fashion for purely altruistic reasons. Thus, it is expected that:

**H12:**  *Altruistic motivation is positively related to the intention to purchase eco-friendly handcrafted products for self-use.*

### 2.5. Consumer Attitudes

Consumer purchases and intentions related to eco-friendly handcrafted products are increasing lately and attitude is a significant factor in determining this trend. Many studies have shown that attitude strongly predicts purchase intention, particularly for sustainable fashion products [26,79]. The environmental and functional values of recycled products also impact consumers’ attitudes [83], especially for those who are repetitive buyers [46]. The impact of attitude on consumer behavior towards the intention to purchase a product as a gift has also been acknowledged [71]. Moreover, the literature suggests that gift items with cultural, emotional, and traditional values have become popular choices among consumers willing to pay more to express gratitude to others. It is therefore expected that:

**H13:**  *Consumer attitudes are positively related to the intention to purchase for gifting.*

Additionally, Liu et al. [84] found that changes in consumer attitudes can affect their purchase intention in clothing consumption. The literature highlights the role of positive attitudes among diversity-seeking consumers and ethnic groups in shaping their responses to products with ethnic orientation [8,37]. It is also suggested that conserving the cultural, social, traditional, and environmental values of ethnically oriented products positively impacts consumers’ attitudes. In line with the findings of Boncinelli, Dominici, Gerini and Marone [38], it is considered that consumers’ attitudes have a positive correlation with the intention to purchase products for personal use. Hence, it is expected that:

**H14:**  *Consumer attitudes are positively related to the intention to purchase for self-use.*

**Figure 1:**
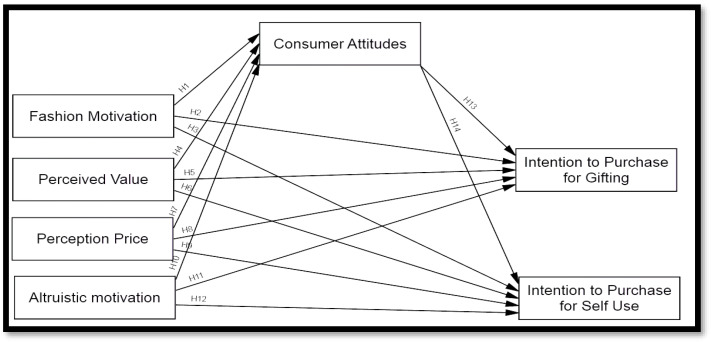
Study Framework.

Based on the set of hypotheses derived from the literature, the conceptual model proposed for this study is presented in Figure 1.

## 3. Materials and Methods

### 3.1. Materials and Measures

The self-administered questionnaire utilized in the study was developed based on the conceptual model presented. It comprised 29 questions concerning eco-friendly handcrafted fashion products made of recycled clothing and the traditional Indonesian fabric batik. The measurement scales were adopted from previous research in the field: three items for Fashion Motivation (FM) were adapted from Aycock [85], four items for Perceived Value (PV) were adapted from Chaturvedi et al. [56], four items for Price Perception (PP) were adapted from Chockalingam and Isreal [72], three items for Altruistic Motivation (AM) and five items for Consumer Attitudes (CA) were adapted from Pop et al. [79], and four items for Intention to Purchase (for gifting and personal use) were adapted from Chaturvedi et al. [56] and Pop et al. [79]. Respondents were asked to rate their answers on a scale of 1 (Strongly Disagree) to 5 (Strongly Agree).

### 3.2. Data Collection and Study Participants

The data for this study were collected through an online survey, to ensure the participation of consumers from various locations. The study focused on eco-friendly handcrafted products made from recycled clothing and traditional Indonesian fabric batik. Data were collected from Indonesian and non-Indonesian consumers using snowball sampling. The survey was conducted in October 2021 and resulted in 289 valid responses.

Of the 289 participants, 39.1 percent were male, and 58.1 percent were female. 33.9 percent were between 21 and 30 years, 28.4 percent were above 40 years old, 23.5 of the respondents were below 20 years old, and 14.2 percent were between 3I and 40 years old. Regarding education, 39.8 percent of the respondents held a bachelor’s degree, 32.2 percent completed up to high school, 25.6 percent held a post-graduation or higher degree, and 2.4 percent did not report their education level. The majority of respondents had a monthly income of below 1000 euros (52.2%), with 28.7% earning between 1001 and 2000 euros, 11.1 percent between 2001 and 3000 euros, 2.4 percent between 3001 and 4000 euros, and 5.5 percent receiving more than 4001 euros. Additionally, 57.1 percent of the participants were from Europe, 33.9 were from Indonesia, and 9 percent reported being neither European nor Indonesian.

## 4. Results

In this study, SPSS 25 was used for the preliminary examination of data. The descriptive analysis presents information about data for all independent and dependent variables in the research study [86]. In addition, the partial least square (PLS) path modeling technique (using the SmartPLS 3.0 software v.3.3.9) was applied to evaluate the validity and reliability of the measurement model and hypotheses testing.

### 4.1. Descriptive, Validity, and Reliability of Measurement Model

As shown in Table 1, the values of observed variables in the proposed models had skewness and kurtosis within the acceptable levels (+2 and −2) [87], except in the case of three items (AM1, IPSU3, and IPSU4) which showed kurtosis higher than the acceptable level (2.784, 2.193, and 2.441 respectively). According to Kline [88], the absolute value of the kurtosis below 0.8 is considered acceptable to prove normal univariate distribution. These results indicated that the data were distributed normally.

The outer loadings are between 0.601 and 0.924, which exceeded the recommended acceptance levels [89]. Hair et al. [90] suggested that the standardized loading for each item should be greater than 0.7 to determine reliability, but a value of 0.5 or 0.6 is still acceptable [89]. Moreover, this study measured the internal consistency reliability by checking composite reliability (CR) [91]. Fornell and Larcker [92] recommended that composite reliability should be greater than 0.7 to be considered adequate. A value greater than 0.7 is regarded as the minimum value for acceptance of Cronbach’s Alpha (α) [93]. As indicated in Table 1, all CR and α are above the recommended range of 0.70. Results show that the convergent validity is supported since the average variance extracted (AVE) exceeded the value of 0.50 [90,91,92].

The heterotrait-monotrait ratio of correlations (HTMT) as a new approach to assess discriminant validity was used in this study. Based on the suggestion by Henseler et al. [94], the HTMT ratio must be less than 0.90 [94]. As shown in Table 2, the HTMT ratios of correlation of each construct are below 0.9, which means the discriminant validity has been established.

### 4.2. Structural Model Evaluation and Hypotheses Testing

**Figure 2:**
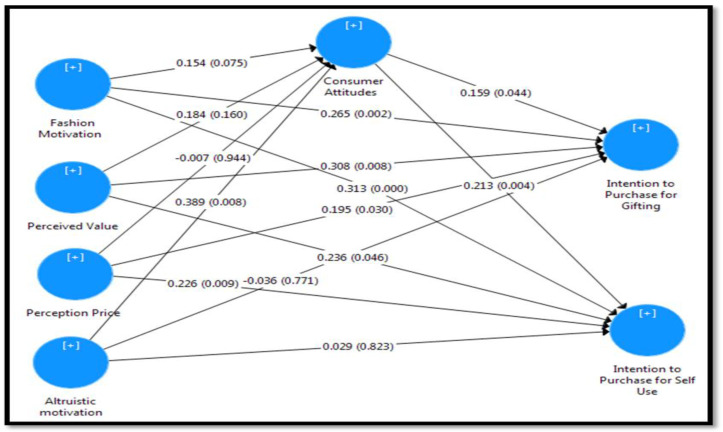
Structural model.

After obtaining the sufficient quality measurement model, the structural model was assessed. According to Chin [89] and Hair et al. [95], the structural model evaluation consisted of the following steps: assessment of the structural relationship in the model for multicollinearity assessment (VIF), coefficient of determination (R^2^), f2 effect size and Q2 predictive relevance and estimation of path coefficients. The results of R^2^, f^2^, VIF, and Q2 are presented in Table 3. The Consistent PLS bootstrapping resampling procedure using 10,000 subsamples and the default settings (i.e., parallel processing, no sign changes) was used to assess the path coefficients and their significance levels (See Figure 2 and Table 3).

Based on the suggestion of Cohen [96], R^2^ values for endogenous latent variables are assessed as follows: 0.26 (substantial), 0.13 (moderate), and 0.02 (weak). As shown in Table 3, the R2 for the endogenous constructs ranged from 0.400 to 0.634. Thus, the R2 for Consumer Attitudes, Intention to Purchase for Gifting, and Intention to Purchase for Self-Use are substantial. In addition, this study used a cross-validated redundancy criterion to examine the predictive relevance (Q2) of the exogenous latent variables on the reflective endogenous latent variable [97,98]. As indicated in Table 3, cross-validated redundancy (Q2) for endogenous latent variables is greater than zero, which indicates that the model is relevant for predicting that factor. To assess collinearity, Variance Inflation Factor (VIF) values were examined as recommended by Hair et al. [90] who suggested that VIF values should be less than 5.0. Table 3 indicates that all exogenous constructs have VIF values less than 5.0, thus indicating no multicollinearity issue in the structural model.

#### 4.2.1. Result of Hypotheses Testing

The results suggest that the effect of Fashion Motivation on Consumer Attitude H1 (β = 0.154, t = 1.778, *p* > 0.05) is non-significant. The results confirm H2 and H3, which predicted a positive effect of Fashion Motivation on Intention to Purchase for Gifting (H2: β = 0.265, t = 3.088, *p* < 0.01) and Intention to Purchase for Self-Use (H3: β = 0.313, t = 3.801, *p* < 0.01). Based on the suggestion of Cohen’s [96] guidelines, the f^2^ values of 0.35, 0.15, or 0.02 for endogenous latent variables in the inner path model are considered large, medium, and small, respectively. Therefore, the impact of Fashion Motivation on Intention to Purchase for Gifting (f2 = 0.086) and Intention to Purchase for Self-Use (f2 = 0.164) are medium and large, respectively.

The direct effect of Perceived Value on Consumer Attitudes (H4: β = 0.184, t = 1.405, *p* > 0.05) was not supported but results show the effect of Perceived Value on Intention to Purchase for Gifting (H5: β = 0.308, t = 2.654, *p* < 0.01) and Intention to Purchase for Self-Use (H6: β = 0.236, t = 1.994, *p* < 0.05) are accepted. The results indicated that the effect of Perceived Value on Intention to Purchase for Gifting and Intention to Purchase for Self-Use is small.

The results show that Perception Price has not influenced Consumer Attitudes (H7: β = −0.007, t = 0.071, *p* > 0.05). In addition, the results show the effect of Perception Price on Intention to Purchase for Gifting (H8: β = 0.195, t = 2.171, *p* < 0.05) and Intention to Purchase for Self-Use (H9: β = 0.226, t = 2.614, *p* < 0.01) are accepted. The results for f2 indicated that the effect of Perception Price on Intention to Purchase for Gifting and Intention to Purchase for Self-Use is small.

As revealed in Table 3, in support of H10, Altruistic motivation has a positive influence on Consumer Attitudes (H10: β = 0.389, t = 2.667, *p* < 0.01). The result of f2 for Altruistic motivation shows a medium effect on Consumer Attitudes. Moreover, the results suggest no significant effect of Altruistic motivation on the Intention to Purchase for Gifting and the Intention to Purchase for Self-Us. Thus, hypotheses H11 (β = −0.036, t = 0.291, *p* > 0.05) and H12 (β = 0.029, t = 0.223, *p* > 0.01) are rejected.

The results confirm H13 and H14, which predicted a positive effect of Consumer Attitudes on Intention to Purchase for Gifting (H13: β = 0.159, t = 2.011, *p* < 0.05) and Intention to Purchase for Self-Use (H14: β = 0.213, t = 2.921, *p* < 0.01). The results for f2 indicated that the effect of Consumer Attitudes on Intention to Purchase for Gifting and Intention to Purchase for Self-Use is small.

#### 4.2.2. Multi-Group Moderation Analysis

This study conducted a partial least squares multi-group analysis (MGA) moderation test to examine the differences between Indonesian and non-Indonesian respondents. The test utilized the full model and all the hypothesized paths. The results of the multi-group analysis for the model with Indonesian and non-Indonesian respondents are presented in Table 4.

As indicated in Table 4, the analysis reveals statistically significant differences between Indonesian and non-Indonesian respondents regarding the influence of perceived value on consumer attitudes toward sustainable fashion products. Furthermore, significant differences between the two groups in the impact of price perception on attitudes and the intention to purchase for self-use were observed. No additional significant differences were found between the two groups.

## 5. Discussion

The results showed that fashion motivation (H1) had no significant effect on consumer attitudes. However, fashion motivation was found to have a significant positive impact on the intention to purchase for gifting (H2). This finding aligns with prior research that suggests that fashion motivation may play a crucial role among consumers when purchasing eco-friendly handcrafted products as gifts [33,34,35]. In addition, this study (H3) also supports the notion that fashion motivation has a significant positive effect on the intention to purchase for self-use, which is consistent with previous studies [33,38] that highlight the importance of fashion motivation as a factor influencing consumer behavior when purchasing eco-friendly handcraft products for personal use [10,85].

Perceived value was found to not significantly influence consumer attitudes (H4). However, when considering the two subsamples separately, it was observed that perceived value had an impact on the attitudes of foreign consumers (i.e., non-Indonesians). Furthermore, the perceived value was found to have a significant influence on the intention to purchase for gifting (H5) and the intention to purchase for self-use (H6). These findings aligned with previous studies [48,54,56,57] that suggest that perceived value is a significant determinant of consumer purchase intentions and behavior. Perceived value is arguably one of the most critical determinants of intention to purchase and the most important concept for understanding the consumer’s mind. Our results imply that consumers are willing to purchase eco-friendly handcrafted products for both gifting and personal use if they perceive the product to be of reasonable value and quality to other products on the market.

The results indicated that, in general, price perception did not have a significant impact on consumer attitudes (H7). However, a significant impact was detected within the Indonesian subsample. Additionally, price perception was found to significantly influence the intention to purchase for both gifting (H8) and self-use (H9). However, the impact of price perception on the intention to purchase for self-use was not significant among foreign consumers (e.g., non-Indonesians). These findings align with existing literature that suggests that price expectations play a role in purchasing decisions [26,64,72]. However, differences can be expected due to cultural factors and the effect of average purchasing power and currency strength. Cultural bonds can dilute the impact of price perceptions on the attitudes of national consumers (in this case, Indonesians). The effect of average purchase power and currency strength (e.g., Euro vs. Rupiah) may also influence the impact of price perceptions on intentions to purchase for personal use.

Altruistic motivation was found to significantly impact consumer attitudes (H10), which supports the positive relationship between altruistic motivation and consumer attitudes found in previous research [79]. However, altruistic motivation was not found to significantly impact purchase intentions for either gifting (H11) or self-use (H12), contradicting the literature that suggests the positive influence of altruistic motivation on sustainable consumption for gifting [48,81] and for personal use [82].

Consumer attitudes were found to significantly impact the intention to purchase for both gifting (H13) and self-use (H14), aligning with the findings of Suarez, Hugo and Paris [71] and Boncinelli et al. [38]. These findings are also consistent and other research on sustainable fashion consumption [26,79]. Connecting the finding with other studies suggests that attitude is an effective predictor of purchase intention in sustainable fashion consumption.

The comparison between Indonesian and non-Indonesian respondents revealed that, apart from three hypothesis paths (perceived value to consumer attitudes, price perception to consumer attitudes, and price perception to intention to purchase for self-use), there was no moderating effect on the other hypothesized paths. In this regard, the Mindsponge Theory [99] and the Mindspongeconomics [100] can offer insights into the different mindsets and priorities that explain divergences in individual reactions regarding eco-friendly handcrafted fashion products. These theories suggest that the flow of information influences mental processes and behaviors, with consumers needing access to information for knowledge and skill development. These processes shape expectations, perceived benefits, and attitudes [101], and consequently can explain variations in attitudes, the dilution of the attitude-purchase intention relationship, and differences between national and foreign consumers.

## 6. Conclusions

This study aimed to explore the factors that determine consumer intention to purchase eco-friendly handcrafted products made from recycled and traditional cloth. The research investigated the impact of fashion motivation, perceived value, price perception, and altruistic motivation on consumer attitudes and their intention to purchase for gifting and self-use. The study also compared Indonesian and non-Indonesian respondents in terms of all hypotheses’ paths.

This research adds to the current understanding of consumer behavior and intention to purchase in the sustainable fashion industry. It highlights the significance of fashion motivation, perceived value, price perception, and altruistic motivation in consumer behavior and decision-making related to eco-friendly products. Hence, it provides a comprehensive examination of the factors that influence consumer attitudes toward such products. Overall, the study provides valuable insights into the factors that influence consumer intention to purchase eco-friendly handcrafted products, contributing to the body of knowledge in the fields of consumer behavior and sustainability marketing and management. Furthermore, the study sheds light on possible differences in behavior regarding the goal of the consumer (personal use or gifting) and the cultural bond with the product identity (i.e., national vs. foreign consumers). By contributing to the advancement of theoretical knowledge in this area, the article advances the field and provides a foundation for further research in the future.

The results of this study and previous research [14,15,102] indicate that managers need to understand consumer behavior across different cultures to make effective marketing strategies or manage customer requirements from various nationalities/cultures. The findings indicate that consumer intention to purchase for gifting and self-use can be influenced by fashion motivation, perceived value, price perception, and consumer attitude. Thus, managers and entrepreneurs should focus on product innovation, particularly fashion trends, to match consumer fashion motivation. Additionally, they should emphasize the environmental friendliness of the product to increase perceived value, which in turn can boost product usage and gifting. The higher price of this type of product should be associated with its quality and environmental impact. This information should be emphasized in marketing communications, regardless of the purpose of the purchase, i.e., personal use or gifting. The results also indicate that managers need to consider cultural differences when developing marketing strategies and managing customer requirements from different nationalities/cultures.

The findings of this study also have policy implications. Social marketing campaigns can play a role in promoting consumer education and awareness of sustainable products, including eco-friendly handcrafted fashion. Policymakers can support small entrepreneurs by incentivizing the adoption of sustainable practices and providing resources and training that help develop innovative fashion products catering to various consumer segments.

This study is not without limitations. This study is limited by its non-probabilistic sampling technique using the snowball method and its application to only one country. In terms of the sample size, although sufficient for the analysis using SmartPLS [103,104], larger sample sizes may provide more robust results. Additionally, the findings indicate that fashion motivation, perceived value, and price perception have no impact on consumer attitudes toward eco-friendly products. Still, these results may not represent the views of all consumers. Further research with larger sample sizes and alternative methods could enhance the understanding of consumer attitudes toward eco-friendly products and sustainable fashion consumption behaviors. This study only investigated intentions to purchase eco-friendly handcrafted fashion products, not actual purchases. Future research could explore the relationship between intentions and behavior.

To consider the cultural nature of traditional eco-friendly handcrafted products, it is suggested that future research examine other cultural cloth materials and fashion craft techniques. Comparisons between cultural backgrounds and products, as well as between the intentions and behaviors of different consumer generations, are also recommended. Demographic variables such as education, human values, and income could also be further explored in future studies.

## Figures and Tables

**Table 1:** Descriptives, validity, and reliability of measurement model (n = 289).

Construct	Items	Mean	SD	Skewness	Kurtosis	Outer Loadings	α	CR	AVE
Fashion Motivation	FM1	3.42	1.091	−0.398	−0.405	0.681	0.794	0.795	0.571
	FM2	3.57	1.022	−0.449	−0.233	0.628			
	FM3	3.73	0.941	−0.671	0.454	0.924			
Perceived Value	PP1	3.46	0.901	−0.554	0.166	0.760	0.922	0.923	0.75
	PP2	3.73	0.86	−0.670	0.676	0.601			
	PP3	3.74	0.888	−0.553	0.220	0.849			
	PP4	3.62	0.893	−0.595	0.473	0.785			
Perception Price	PV1	3.7	0.855	−0.697	1.178	0.838	0.838	0.839	0.569
	PV2	3.78	0.871	−0.754	1.050	0.876			
	PV3	3.71	0.911	−0.924	1.147	0.858			
	PV4	3.73	0.88	−0.923	1.364	0.891			
Altruistic motivation	AM1	4.04	0.824	−1.278	2.784	0.798	0.838	0.838	0.634
	AM2	3.99	0.837	−0.688	0.454	0.754			
	AM3	3.9	0.834	−0.643	0.613	0.835			
Consumer Attitudes	CA1	4.18	0.842	−0.942	0.953	0.687	0.861	0.857	0.547
	CA2	4.23	0.744	−0.861	1.003	0.636			
	CA3	4.35	0.691	−0.970	1.497	0.735			
	CA4	4.35	0.763	−1.111	1.204	0.791			
	CA5	4.15	0.884	−0.849	0.252	0.832			
Intention to Purchase for Gifting	IPG1	3.56	0.923	−0.556	0.237	0.836	0.924	0.924	0.752
	IPG2	3.75	0.811	−0.897	1.410	0.872			
	IPG3	3.78	0.828	−1.018	1.852	0.849			
	IPG4	3.8	0.835	−0.897	1.456	0.911			
Intention to Purchase for Self-Use	IPSU1	3.36	0.842	−0.366	0.278	0.797	0.854	0.854	0.595
	IPSU2	3.68	0.818	−0.732	1.180	0.756			
	IPSU3	3.87	0.761	−1.009	2.193	0.737			
	IPSU4	3.83	0.822	−1.148	2.441	0.793			

Notes. Cronbach’s Alpha (α); Composite Reliability (CR); Average Variance Extracted (AVE), Std. Deviation (SD).

**Table 2:** Results of Discriminant validity.

Construct	1	2	3	4	5	6	7
1. Altruistic motivation							
2. Consumer Attitudes		0.598					
3. Fashion Motivation		0.603	0.457				
4. Intention to Purchase for Gifting		0.528	0.489	0.530			
5. Intention to Purchase for Self-Use		0.620	0.579	0.621	0.895		
6. Perceived Value		0.683	0.509	0.466	0.628	0.673	
7. Perception Price		0.489	0.367	0.368	0.554	0.602	0.710

**Table 3:** Results of structural model evaluation and hypotheses testing.

Direct Path	Beta	T Value	*p* Value	*f* ^2^	VIF	Results
Fashion Motivation → Consumer Attitudes	0.154	1.778	0.075	0.025	1.598	Rejected
Fashion Motivation → Intention to Purchase for Gifting	0.265	3.088	0.002	0.086	1.638	Accepted
Fashion Motivation → Intention to Purchase for Self-Use	0.313	3.801	0.000	0.164	1.638	Accepted
Perceived Value → Consumer Attitudes	0.184	1.405	0.160	0.020	2.876	Rejected
Perceived Value → Intention to Purchase for Gifting	0.308	2.654	0.008	0.065	2.933	Accepted
Perceived Value → Intention to Purchase for Self-Use	0.236	1.994	0.046	0.052	2.933	Accepted
Perception Price → Consumer Attitudes	−0.007	0.071	0.944	0.000	2.041	Rejected
Perception Price → Intention to Purchase for Gifting	0.195	2.171	0.030	0.037	2.042	Accepted
Perception Price → Intention to Purchase for Self-Use	0.226	2.614	0.009	0.068	2.042	Accepted
Altruistic motivation → Consumer Attitudes	0.389	2.667	0.008	0.108	2.320	Accepted
Altruistic motivation → Intention to Purchase for Gifting	−0.036	0.291	0.771	0.001	2.571	Rejected
Altruistic motivation → Intention to Purchase for Self-Use	0.029	0.223	0.823	0.001	2.571	Rejected
Consumer Attitudes → Intention to Purchase for Gifting	0.159	2.011	0.044	0.031	1.666	Accepted
Consumer Attitudes → Intention to Purchase for Self-Use	0.213	2.921	0.004	0.074	1.666	Accepted
		R Square		Q2		
Consumer Attitudes		0.400		0.191		
Intention to Purchase for Gifting		0.503		0.345		
Intention to Purchase for Self-Use		0.634		0.348		

**Table 4:** Multi-group hypothesis testing.

Direct Path	Path Coefficient			T Value	*p* Value
	Indonesian	Non-Indonesian	Difference		
Fashion Motivation → Consumer Attitudes	0.244	0.107	0.136	1.027	0.305
Fashion Motivation → Intention to Purchase for Gifting	0.164	0.216	0.052	0.358	0.720
Fashion Motivation → Intention to Purchase for Self Use	0.148	0.27	0.121	0.988	0.324
Fashion Motivation → Consumer Attitudes	0.244	0.107	0.136	1.027	0.305
Perceived Value → Consumer Attitudes	−0.026	0.343	0.369	2.032	0.043
Perceived Value → Intention to Purchase for Gifting	0.127	0.387	0.260	1.545	0.123
Perceived Value → Intention to Purchase for Self Use	0.170	0.288	0.118	0.718	0.473
Perception Price → Consumer Attitudes	0.224	−0.092	0.315	2.143	0.033
Perception Price → Intention to Purchase for Gifting	0.312	0.136	0.176	1.221	0.223
Perception Price → Intention to Purchase for Self Use	0.388	0.093	0.295	2.308	0.022
Altruistic motivation → Consumer Attitudes	0.201	0.305	0.104	0.532	0.595
Altruistic motivation → Intention to Purchase for Gifting	0.163	−0.041	0.205	1.285	0.200
Altruistic motivation → Intention to Purchase for Self Use	0.149	0.067	0.082	0.502	0.616
Consumer Attitudes → Intention to Purchase for Gifting	0.068	0.159	0.091	0.713	0.476
Consumer Attitudes → Intention to Purchase for Self Use	0.136	0.192	0.055	0.502	0.616

## Data Availability

The data presented in this study are available on request from the corresponding author.
